# Interdisciplinary staff perceptions of advance care planning in long-term care homes: a qualitative study

**DOI:** 10.1186/s12904-022-01014-2

**Published:** 2022-07-15

**Authors:** Shirin Vellani, Elizabeth Green, Pereya Kulasegaram, Tamara Sussman, Abby Wickson-Griffiths, Sharon Kaasalainen

**Affiliations:** 1 Faculty of Health Sciences, School of Nursing, McMaster University, 1280 Main Street West, Hamilton, ON L8S 3Z1 Canada; 2 Faculty of Arts, School of Social Work, McGill University, 3506 University St, Montreal, QC H3A 2A7 Canada; 3 Faculty of Nursing, University of Regina, 3737 Wascana Parkway, Regina, SK S4S0A2 Canada

**Keywords:** Advance care planning, Long-term care home, Interdisciplinary communication, Palliative approach

## Abstract

**Background:**

Residents living in long-term care homes (LTCH) have complex care needs, multiple chronic conditions, increasing frailty and cognitive impairment. A palliative approach that incorporates advance care planning (ACP) should be integrated with chronic disease management, yet it is not a norm in most LTCHs. Despite its growing need, there remains a lack of staff engagement in the ACP process.

**Objectives:**

The aim of this study was to explore the perceptions and experiences of interdisciplinary staff related to the practice of ACP in LTCHs.

**Methods:**

This study is part of a larger Canadian project, iCAN ACP, that aims to increase uptake, and access to ACP for older Canadians living with frailty. An exploratory qualitative design using an interpretive descriptive approach was employed utilizing focus groups and semi-structured interviews with staff from four LTCHs in Ontario, Canada.

**Findings:**

There were 98 participants, including nurses (*n* = 36), physicians (*n* = 4), personal support workers (*n* = 34), support staff (*n* = 23), and a public guardian (*n* = 1). Three common themes and nine subthemes were derived: a) ongoing nature of ACP; b) complexities around ACP conversations; and c) aspirations for ACP becoming a standard of care in LTCHs.

**Discussion:**

The findings of this study provide important contributions to our understanding of the complexities surrounding ACP implementation as a standard of practice in LTCHs. One of the critical findings relates to a lack of ACP conversations prior to admission in the LTCHs, by which time many residents may have already lost cognitive abilities to engage in these discussions. The hierarchical nature of LTCH staffing also serves as a barrier to the interdisciplinary collaboration required for a successful implementation of ACP initiatives. Participants within our study expressed support for ACP communication and the need for open lines of formal and informal interdisciplinary communication. There is a need for revitalizing care in LTCHs through interdisciplinary care practices, clarification of role descriptions, optimized staffing, capacity building of each category of staff and commitment from the LTCH leadership for such care.

**Conclusion:**

The findings build on a growing body of research illustrating the need to improve staff engagement in ACP communication in LTCHs.

**Supplementary Information:**

The online version contains supplementary material available at 10.1186/s12904-022-01014-2.

## Background

The majority of residents living in long-term care homes (LTCH) have complex care needs, multiple comorbid conditions, increasing frailty and some form of cognitive impairment [1–3]. In Canada, in a given year, 52% of individuals living in LTCHs will die [1, 3] and the number of deaths in LTCHs is expected to rise as increasing numbers of residents remain there until the end of their lives [1, 4–6]. Due to the growing complexities in care requirements, and the fact that LTCHs are now a significant site of death, a priority needs to be given to ensuring high quality of care is provided to residents and their families that meet their wishes throughout their illness trajectory including at the end-of-life (EOL) [2, 7].

Advance care planning (ACP) is a process that supports people in reflecting on and sharing their personal values, life goals, and preferences related to future care [7, 8]. The goal of ACP is to prepare a person, and their proxy decision-maker(s), for future care decisions, so that they receive care that is consistent with their values and preferences during serious illness [7, 8] including at the EOL. Prior ACP discussions should help proxy decision makers and staff make decisions about current care (e.g., worsening stage of dementia, hospitalization and discussion about major clinical procedures) as well, at EOL should a resident lack capacity to participate [9]. As such, ACP is a central component of a palliative approach to care [10–12]. ACP is related to goals of care communication. While ACP focuses on future care, goals of care communication can include the person or only family (if resident is not capable) and focuses on specific treatment preferences [13]. As such, prior ACP discussions should inform goals of care communication with residents and/or their care partners related to residents’ current clinical circumstances.

With the potential to bridge incongruencies between the residents’ wishes and the care they actually receive, it is essential that LTCH staff incorporate a palliative approach to care that integrates ACP for residents [14]. Patient-provider ACP communication has been shown to improve the patient experience, align treatment with patients’ preferences, avoid unwanted and costly invasive treatments near EOL, and improve psychological outcomes for family members related to proxy decision making and during bereavement [7, 15–20]. Despite the growing needs and benefits of ACP for LTCH residents and their families, this practice remains rare in LTCH environments in Canada and abroad [21]. Research indicates there is a lack of staff engagement in discussions related to ACP including wishes for EOL care in LTCHs [3, 22]. Lack of sufficient knowledge, skills, ability, and time to participate in ACP, and inadequate administrative systems in place have all been documented barriers to ACP conversations in LTCHs [16, 23–27]. In addition, there is lack of clarity as to the staff responsible for initiating the ACP discussions with residents and their family care partners [28], who not only play essential role in assisting staff with routine personal care and recreation but make care decisions when residents have compromised or lost decision making capacity [29, 30].

This study is part of a larger Canadian project, iCAN ACP, that aims to increase uptake, and access to ACP for older Canadians living with frailty across the primary care, LTCH and hospital sectors. This study was initiated to inform the LTCH sector of the project. While literature is beginning to emerge on the challenges and potential solutions regarding ACP engagement in LTCH much of the research is focused on nurses and nursing aides with far fewer studies representing the range of staff who typically provide and oversee care to residents such as dietary aides and recreational staff. If all staff in LTCH are to play a role in ACP provision than the voices of all staff should be included in research. The aim of this paper is to explore the interdisciplinary staff’s (e.g., clinical staff, support staff, and appointed guardians) perceptions and experiences regarding ACP discussions with residents and care partners. Exploring key stakeholders’ experiences with ACP engagement is an essential step in developing and successfully implementing tailored ACP tools and programs in the LTC sector and ensuring anticipated benefits are realized [31–33].

## Methods

### Design

We designed an exploratory qualitative study to explore participants’ accounts related to the practice of ACP in their respective LTCHs. We used an interpretive descriptive approach because it is well suited for capturing knowledge and experiences related to a clinical phenomenon in order to highlight what exists and what needs to be done to impact clinical practice [34]. We used focus groups and individual interviews as methods of data collection. We employed both strategies to ensure broad based participation and support the development of a comprehensive view of the phenomena (i.e. ACP) [35–37]. For example, physicians and a public guardian (PG) participated in individual interviews, as they could not attend a focus group due to scheduling conflicts given, they work in multiple places. Using combined methods of data collection is supported by interpretive description [33]. We conducted the research in accordance with the standards of the Tri-Council Policy Statement for Ethical Conduct for Research Involving Humans 1998 (with 2000, 2002, and 2005 amendments) [38]. Procedures were approved by the Office of Research Ethics Board at McGill and McMaster Universities. All staff provided informed consent to participate in this study. We followed the Standards for Reporting Qualitative Research guidelines throughout the study process [35, 39].

### Participants and setting

Focus groups and interviews were conducted with staff members at four LTCHs in Southern Ontario. These LTCHs were selected as they were representative of the contexts seen in homes across Canada in terms of ownership (for profit, not for profit) and size (number of beds ranging from < 100 to > 150). The size and ownership have been found to have impact in successful enactment of a new initiative [40]. Maximum variation sampling was used by recruiting various categories of staff to gather rich information on prevailing and distinctive perspectives on ACP in the context of LTCHs [36]. Participants were recruited to partake in focus groups or interviews using posters in the LTCHs and invitation via management teams of the LTCHs. Various categories of LTCH staff were recruited to acquire understanding of multiple individuals’ relatable and diverse experiences and perspectives within a broader phenomenon of ACP practice in LTCHs [41]. LTCH staff who worked on site in full-time capacity or periodically and who interacted with residents about their care needs and preferences were eligible to participate.

### Data collection

A total of eleven profession specific focus group sessions were conducted separately with personal support workers (PSWs) (*n* = 34), nurses (*n* = 36) and support staff from four LTCH (*n* = 24). The number of participants in each focus group ranged from three to 12, lasting for 38 min on average. Separate sessions were conducted to provide opportunity to each staff category to openly share their discipline specific roles and responsibilities, as well as perspectives that may result in diversity of experiences with ACP without feeling judged by the others. This was necessary in light of work hierarchies and strict divisions of task existing in Canadian LTC homes resulting in disproportionate power distribution and prejudiced social relations between different categories of staff [42]. Also, previous research has mostly focused on nurses’ perspectives or grouped HCPs together, failing to account for the individual discipline related experiences, challenges and/or viewpoints [43–46] and recommendations. Additional file 1 provides an overview of the functions for each category of staff in the context of Ontario, Canada.

A focus group guide was developed to stimulate thoughts, reflections, and discussion about the following areas: opinion on ACP in LTCHs, current practices, their role, and challenges in implementing ACP (See Additional file 2 for the focus group guide). Sessions were facilitated by two members of the research team, one of whom also recorded field notes during the sessions to collect observational data [47]. Specifically, field notes were recorded on focus group dynamics, non-verbal cues, emerging questions or ideas and any other relevant observations [48]. This information supported the verification of transcriptions and data analysis as they provided contextual data.

One physician from each LTC facility (*n* = 4) and a PG were also recruited who participated in one-on-one audio recorded interviews. Two semi-structured interview guides were developed; one for physician interviews and a second one for the PG interview to capture their profession and role specific perspectives on same topics as the focus group. Each physician participated in one interview lasting for 30 min on average. Only one PG expressed interest in participating in this study and was interviewed (33 min). They provided service in one inner city home that had a large population of residents who were once homeless before moving into LTCH. And staff at this site worked closely with the PG due to a lack of family involvement and was interested to participate. We felt that this was a unique experience; hence, we explored this role in an in-depth manner. All focus group sessions and interviews were conducted by a research coordinator with an undergraduate education and had training in qualitative data collections methods. While a research assistant, who is a nurse by training, served as a note-taker to keep track of the topics discussed. Both were supervised by faculty who had extensive experience in interviewing and qualitative methods. Regular meetings were held during the interviewing process to review transcripts, reflect on them, and discuss ways to respond to emerging themes and probe in subsequent interviews. The interviewer and the note taker were part of the research team and had no prior relationship with the participants.

### Data analysis

Each focus group was audio recorded and transcribed verbatim by an external transcriber who also anonymized any identifying information. Transcriptions were reviewed by a member of the research team (PK) for accuracy prior to data analysis. Dedoose, a web-based qualitative analysis program was used to manage, organize, and code the data; as well, help track the analysis process. Inductive thematic analysis was utilized for data analysis as an in-depth method for exploring LTCH staffs’ experiences related to ACP in their practice [49]. The analysis was conducted in five steps: familiarization with the data; development of initial codes; clustering of key codes into tentative categories; collective reflections on codes and categories to inform the development of themes; and refining and renaming final themes through the process of writing and continued reflection [50]. In the first stage each transcript was read and re-read by (PK) and (EG) who independently developed initial codes thought to broadly capture participants’ thoughts, experiences, and reactions to ACP, what Braun and Clarke refer to as reflexive thematic analysis [51]. In the second stage PK and EG discussed and reflected on their initial coding and collectively developed a series of descriptive codes thought to comprehensively address the research questions. In the third stage PK and EG reviewed all descriptive codes and their associated excerpts looking for patterns between and across codes. Through the combination of independent review, reflection and discussion descriptive codes were placed under tentative categories. In the fourth stage, PK, EG, SV, TS and SK engaged in discussions and reflections about the codes, and categories generated thus far. The team also engaged in comparing different participants’ accounts within and between one another incorporating data source triangulation and helped enhance our understanding of the phenomenon [52]. The team's collective expertise in nursing, social work, LTC and end of life communication served to be instrumental in the analysis process. Field notes and the teams’ knowledge of the literature also informed these discussions. This process resulted in the development of themes that were used to form the foundation of a report that was written by PK and reviewed and refined by EG. In the fifth and final stage the report was circulated to all co-authors who reviewed and refined the themes based on their collective knowledge of the literature on ACP, LTC and interdisciplinary practice and a careful review of the coded extracts. As such, the full research team engaged in discussing the data from their unique perspectives and arrived at a shared understanding adding depth to the process (investigator triangulation). The research team participated in collective reflexivity through nuanced discussions and reflections on our own thoughts and assumptions related to ACP in the context of LTC residents and their impact on the interpretation of the data [53].

We ensured rigorous trustworthiness and credibility of the findings through holding debriefing sessions with the research team; practicing researcher reflexivity as well as triangulation; maintaining a detailed record of the analysis process including decision notes; systematically managing data and keeping an exhaustive record of the process and decisions made; managing data systematically; and reviewing different accounts [54]. The research team reviewed the full inventory of themes and reached consensus on three broad categories and ten subcategories. The team also reviewed and read each subcategory in relation to the coded data to optimize internal consistency and refine them as appropriate. It was also determined that the categories were cogent, and at the same time, significantly distinct to render a story captured in the data [49]. Subsequently, names of final categories and subcategories were created by the team [49, 50], and presented in Table 2.

## Findings

A total of *n* = 98 staff from four LTCHs participated in this study, and included PSWs (*n* = 34), nurses (*n* = 36) and support staff (*n* = 23), physicians (*n* = 4) and a PG (*n* = 1) (See Table 1 for participant characteristics). Of the four homes, three operated on a for-profit while the other on a not-for-profit basis. The number of beds ranged from 64–206 per LTCH. Three themes were derived in relation to the perspectives of LTCH staff on ACP for residents they cared for. Themes were common between all categories of staff and included: a) ongoing nature of ACP; b) complexities around ACP conversations; and c) aspirations for ACP becoming a standard of care in LTCHs across all disciplines. The themes and subthemes are presented in Table 2 and detailed below with most representative quotes presented in the text.

**Table 1:** Characteristics of participants and LTC

	Nurses (*n* = 36)	PSW (*n* = 34)	Physicians (*n* = 4)	SS (*n* = 23)	PG (*n* = 1)	TOTAL *N* = 98
**Age**, Mean (SD)	43.5(8.8)	48.0 (10.6)	57.0(3.2)	41.5(14.4)	-	44.1(11.5)
**Gender, % Females**	28(77.8)	32 (94)	2(50)	18(78.0)	0	80(81.6)
**Employment status n (%)**						
Part time	9(25)	8(23.5) ^a^	4(100)	7(30.4)	1(100)	29 (29.5)^a^
Full time	26(72.2)	24(70.5)		19(82)		69(70.4)
**Length of time working in LTC (years), Mean (SD)**	9.9(8.1)	15.1(9.5)	10.0(4.6)	11.2(9.0)	8	11(8.9)
**Prior palliative training, n (%)**	18(51.6)	25(73)	2(50)	10(37.5)	1(100)	56(57.1)

^a^% may not equal 100 due to missing responses

**Table 2:** Themes and subthemes related to LTCH staff perceptions on ACP

Themes	Subthemes
Ongoing nature of ACP	- Right time to initiate ACP discussions is when the person can participate
	- ACP discussions can make transition to EOL more seamless
	- ACP should be holistic to guide EOL care
Complexities around ACP conversations	- Identifying residents’ values and wishes when no former ACP
	- Navigating divergent resident and family perspectives
	- Staff’s lack comfort to broach ACP conversations
Aspirations for ACP becoming a standard of care in LTCHs across all disciplines	- Prioritizing ACP as a standard of care
	- Need for training and capacity-building for staff to support residents and families in ACP
	- From hierarchies to building a team approach

### a) Ongoing nature of ACP

This theme highlights the staff’s appreciation of the dynamic nature of the ACP process in terms of it being fluid and productive. Many expressed their thoughts on the idea of the right time to initiate the ACP conversations while also appreciating that it may involve multiple interactions depending on the residents’ and family care partners’ level of readiness. Participants shared their perceptions about what should be included in ACP discussions and that ACP conversations should be comprehensive to guide residents’ care discussions when they experience acute changes in their condition such as transitioning to end-of-life or losing decisional capacity.

#### Right time to initiate ACP discussions is when the person can participate

In relation to the right time, many staff shared their awareness of the importance of timing in initiating ACP discussions and that these discussions should commence when the residents have the cognitive capacity to partake in it. Some staff noted that the ACP process should begin long before the admission into a LTCH, highlighting that residents are generally admitted in the late stages of their disease trajectory. By which time, many may have lost their cognitive abilities to engage in ACP. One nurse explained:

It’s very important to be able to say your wishes while you’re able to. You get diagnosed with Dementia, somebody should discuss the trajectory of the disease and what the next steps are and what you need to look forward to…I mean you have Dementia, that’s the end of the discussion… it really has to be done long before Long Term Care of today anyway. Because the residents are coming in much sicker, much more deteriorated than they used to. (Nurse site 1)

Staff also acknowledged that ACP discussions can be difficult due to multifactorial reasons, such as cultural differences, worries about causing hopelessness, lack of staff confidence and lack of readiness and capacity in residents/care partners. Some staff also articulated the importance of leveraging those moments when residents with cognitive impairment may present with periods of lucidity and their care wishes could be elicited. Staff mutually communicated the importance of capturing the opportunities of holding ACP conversations to learn more about residents’ reflections and wishes. Awareness of residents’ wishes, and values can also better prepare their family care partners for what to expect with the progression of the disease/s, alleviate their psychological distress, and prepare for in the moment proxy decision-making. One support staff described:

It makes it easier on us here if we have an idea as to what is going to happen to the person, the person’s wishes and everything. And if nobody knows and the person is here now, we have to start playing a guessing game after. (Support Staff site 4)

As such, LTCH staff acknowledge that the right time to begin ACP process is when the person can meaningfully participate in it, which may be prior to admission to the home. When possible, staff engage in ACP with residents, which can guide goals of care discussions as their illness (es) advance and/or they lose cognitive capacity to communicate for themselves.

#### ACP discussions can make transition to EOL more seamless for residents and families

While some participants spoke of ACP as a static formulaic plan, many more recognized the fluid conversational and dynamic nature of ACP. Many highlighted the importance of pivoting the conversation to goals of care discussions (e.g., use or non-use of life sustaining treatments in current clinical context), as well, wishes for terminal and EOL care with residents and family care partners when there is acute changes in condition, general deterioration of health, when no beneficial treatment options are realistic and/or residents have declined cognitive capacity for decision making. One physician explained:

So once the person is at that stage and we’ve done everything possible, the patient’s family usually have a lot of questions and you have to be ready to respond to all those questions. The most important thing is we have to explain that we have done…like this is not just giving up but we have made every possible way to make the residents life easier and be pain free and you know in the palliative care that’s the end goal of treatment, too. (Physician site 3)

In cases where residents lose their capacity for decision making, the role of family care partners and in some cases, PG was highlighted. Many staff identified that prior ACP discussions make “in-the-moment” crisis decision making easier for family care partners and prevent the feelings of guilt and psychological distress in them. Several staff members also pointed out that many residents are admitted to LTCHs with no prior ACP discussions, and they can no longer engage in ACP (e.g., due to advanced dementia). In this case, staff engage in goals of care discussions with their family care partners and proxy decision makers with changes in condition to discuss specific treatments. Staff, generally nurses and physicians, not only share residents’ overall status and prognosis but also attempt to learn more about the resident as a person, including their values, goals and wishes. This nurse conveyed their experience in such cases as follows:

And that’s the important thing when we talk to families is to remind them. You may not have had the discussion in the last year but throughout that person’s life have they ever said anything to you about, I don’t want to be kept alive by machines. Like those kinds of conversations. You have to sometimes help them recall those events in their lives. (Nurse site 3)

Nonetheless, several staff identified that when it comes to the PG making the care decisions, most of them generally opt for full code, and not necessarily a holistic palliative care at the EOL. In essence, lack of prior engagement in ACP and awareness of residents’ wishes can impede seamless transition to person-centered EOL care.

#### ACP should be holistic to guide EOL Care

The dynamic nature of ACP was also prominent in relation to it being a productive activity. Many staff noted that ACP discussions should involve holistic discussions about persons’ wishes for all aspects of care they may want near the EOL. Having this knowledge can increase the likelihood of receiving wish concordant EOL care and a better death experience for residents, family, and staff. Several staff iterated that ACP discussions should provide opportunities to learn about residents’ range of preferences including views on life sustaining treatments such as artificial nutrition and mechanical ventilation; religious and spiritual traditions such as how to manage a deceased body and social preferences such as who a resident may want around them (if anyone) in the last days/hours of life. Some staff shared a new initiative implemented in their LTCH called, “My Wishes” whereby, recreational staff engage residents in discussions on their concerns, wishes and hopes for personalized EOL care. As a result, staff were able to acquire a more holistic view of what ACP can entail. One participant expressed:

We do always or try to always think about someone’s spiritual needs at the end of life. So if we’re seeing someone when they’re palliative or end of life we often ask either the doctor or the facilities to consider maybe having a Priest or a Chaplain visit them, or surrounding them with things that they like, or music or things like that” (PG).

Overall, the staff articulated the appreciation for the ongoing nature of ACP process that should commence prior to the LTCH admission or the loss of persons’ cognitive capacity for decision making. Staff do their best to learn about residents in cases they have not had prior ACP discussions to be able to deliver EOL care that is in concordance with residents’ wishes.

### b) Complexities around ACP conversations

The second theme regarding staff’s perception on ACP involving LTCH residents underscored the complexities associated with these discussions. These complexities were in relation to residents who have already lost their cognitive capacities for decision making at the time of LTCH admission, differences in opinions between residents and their family care partners and staff’s lack of comfort to delve into ACP discussions.

#### Identifying residents’ values and wishes when no former ACP

Several staff relayed that many residents are unable to participate in ACP discussions because they have lost their cognitive abilities required for such discussions (e.g. advanced dementia) or are disempowered and not afforded opportunities to engage in ACP. Staff also stated that many of the residents had not previously identified a proxy decision maker or participated in sharing their values and wishes, particularly those, who were either homeless prior to the LTCH admission or did not have any family or friend, automatically become “full code”, and may potentially receive cardiopulmonary resuscitation, which is futile in most cases. Such residents without family or friends are generally served by a PG, who cannot partake in ACP discussions but make other healthcare, shelter, hygiene and safety related decisions required based on individual’s current situation. Many staff articulated that goals of care discussions and completion of level of care form are not the same as ACP, as these frequently involve proxy decision makers and not residents due to their loss of cognitive capacity to participate in these discussions. One nurse explained:

Unfortunately, when they arrive in long term Care, most or a high percentage do not have the capacity to make those decisions…which is why our form is actually not followed within legal parameters because it’s not the resident who is doing the Advanced Care Planning it’s somebody else. So Advanced Care Planning is what I want for me, not what my daughter wants for her. (Nurse site 1)

Staff also expressed that sometimes when residents can make health care decisions, they do not want to “hurt” their families by expressing their true desires for their own care and that can be a source of psychological distress for them. In some cases, residents are not provided an opportunity to express their wishes, as, there is an underlying assumption among family care partners, that residents cannot speak for themselves once admitted to a LTCH. Some staff shared their frustration with this practice. One PSW described it as follows:

… when they are in the nursing home, and they are kind of like under the POA [Power Of Attorney for Personal Care] or the daughter or whatever. So they are not able to get what they want. Most of the time, like they are there, like they are listening to their children…and it’s frustrating because you know they don’t want that but the son or the daughter, they say oh you want this way…and it’s really frustrating. So we don’t know what to do at that time. But the person who is here is really helpless…even though she can talk, but it’s more of the POA. (PSW Site 1)

#### Navigating divergent perspectives

Various staff members expressed the complexities navigating differing resident and family perspectives related to care wishes. For example, there are situations when residents nearing their EOL refuse to eat, but families would request nutritional supplements. In some cases, there are differences of opinions amongst the family members, whereas, in others, some family members emerge after a prolonged absence. A nurse described:

We have some challenges where residents that don’t have family. But then somebody comes at end of life and expects something totally different from what the resident has told us. (Nurse site 2)

In many of these cases staff serve as advocates for residents in front of the families, particularly when they have come to know the residents’ wishes shared during moments of care. Sometimes staff serve as facilitators between residents and families in cases when either may be apprehensive to initiate the discussion. Staff also expressed that frequently when residents’ wishes are unknown, families are conflicted about what might the resident want and/or what would be in their best interest making timely ACP discussions even more important. Staff try their best to bring forth residents’ wishes when known, to facilitate goals of care discussions and other care decisions with the families. It is unclear how often these discussions are successful in devising care plans concordant with residents’ wishes. This support staff explained:

Someone could think oh I think this is their best wish but another child could think something else. So it’s definitely important to talk about and even for the family to sit down and talk about it separately, maybe they want to do that privately and then come back and have another meeting to discuss it again. (Support Staff, site 1)

The above quote is powerful as it alludes to the idea expressed by several other participants suggesting residents may have expressed their desires, but sometimes family gets the precedence to decide. While the differences in opinions pose communication challenges and psychological distress for staff, residents, and care partners, many LTCH staff strive to keep residents wishes at the core in coordinating a care plan. As one nurse described:

If resident comes forth to express their wishes such as for pain control at end-of-life, then staff would inform the family of their wishes, our role in that discussion would be as the advocate for the resident… or to facilitate something between them. For example a mother and daughter example, I said, can I help you talk to your daughter? Is that something you want to do? (Nurse, site1)

Given, prior ACP discussions are not legally binding in Ontario, sometimes it can be hard to implement care in concordance with residents’ shared wishes if families do not agree with them, especially when residents have lost decision-making ability later in their illness trajectory. One physician shared that, “I try to just kind of work with the families all the time, right? To explain the wishes of the patient first and then to give them some statistics or some evidence you know like around the care and everything and what that means.” (Physician site 4).

#### Staff’s lack of comfort to broach ACP conversations

Although many staff members take it upon themselves to engage in conversations related to residents’ values and wishes, several expressed lacking comfort to broach ACP conversations with residents and families. Many staff conveyed their lack of scope of practice to intervene and that they generally redirect requests for ACP and goals of care conversations to charge nurses, physicians and sometimes administrators. PSWs and support staff described feeling a lack of authority or educational preparation to approach ACP with residents and would rather nurses perform this task, yet they develop stronger relationships with residents due to hands-on personal care. One PSW mentioned, “Because I am not qualified, because I’m not a nurse and I’m not a doctor. I don’t feel comfortable having that conversation.” (PSW site 4). Several staff members including PSWs, Registered Practical Nurses (RPN) and Registered Nurses perceived that it is not in their scope to engage in ACP conversations and having such discussions may be misconstrued as “overstepping our boundaries” (RPN site 4). Staff also shared their observations about physicians, where some may not be “open and direct” while others avoid the topic of ACP and palliative care altogether. As such, staff working on-site may have the opportunity but not the authority and confidence to have ACP conversations.

As such, staff expressed the complexities involving ACP conversations in LTCHs related to residents’ lack of cognitive capacity, resident and family dynamics and lack of their own educational preparation and experiences. Yet, staff try their best to bring residents’ wishes to the fore by playing the roles of advocate, educator, and facilitators.

### c) Aspirations for ACP becoming a standard of care in LTCHs across all disciplines

The final theme describes staff’s mutual desire to incorporate ACP in routine care of residents through recognizing ACP as a standard of care, reducing engrained hierarchies and using an interdisciplinary team approach; and concerted efforts to increase the capacity and confidence of each member of the LTCH workforce to delve into ACP conversations with residents and family care partners.

#### Prioritizing ACP as a standard of care

Without exception, staff conveyed the importance of ACP with LTCH residents to better plan for their care as they progress through their illness(es). Based on their experiences, many staff identified that prior ACP discussions make it easier to pivot to goals of care discussions with families when residents have advanced disease or in crisis (e.g., exacerbation of illness, injury, acute illness). ACP better prepares the family for “in-the-moment” decision making that is informed by residents’ wishes as it gives them a “roadmap going forward”. Staff also expressed that having a standard process for ACP can also positively impact families’ experiences with EOL care and bereavement. One nurse explained:

I guess the end of life, no one will know when that moment will happen but when the whole family has good communication and mutual understanding in advance so it will help the process of grieving.” (Nurse site 2)

Staff also identified that having ACP as a standard of care will serve to bring residents, family care partners and staff on the same page in terms of making a collaborative effort to initiating and devising a coordinated care plan informed by residents’ expressed values and wishes.

#### Need for training and capacity-building for staff to support residents and families in ACP

All categories of staff expressed the need and desire to have increased capacity to expand their knowledge, skills, and competence to partake in ACP and goals of care conversations with residents and their family care partners. PSWs and support staff want to be a part of the team when it comes to ACP conversation, as described by this PSW, “I do believe it should involve PSWs because we are the ones that see the residents every day and we’re the ones that actually sit and talk to the residents and the majority of the residents recognize our faces because that’s who is with them.” (PSW site 3). Participants viewed ACP as both formal or informal conversations, some occurring during prebooked family care meetings while others were unplanned and informal such as while reading with a resident in their room or providing personal care. Many staff members appreciated that it is important to be prepared to engage in ACP discussions as they may occur at any time.

Although regulated staff appreciated the relationships that PSWs form with residents due to being most intimately involved in their care, they expressed apprehension for PSWs engaging in ACP conversations due to their lack of training. Some staff who have completed training such as Learning Essential Approaches to Palliative care (LEAP) find it instrumental in facilitating ACP conversations. Several staff voiced that previously implemented chronic illness trajectory resources supporting a palliative approach to care (such as for dementia, frailty, heart failure, lung, and kidney diseases) have empowered them and helped decreased their feelings of discomfort with ACP conversations with residents and family care partners. They agree that training and resources should be available for all categories of staff including PSWs, physicians, external consultants and family care partners. One participant also described the utility of illness specific ACP resources for family care partners as follows:

I think there were six of them … there was the Alzheimer’s, there was the COPD, there was a bunch of them… So that was really good because then people, even though they’ve gone to the doctor a few times they might not know the whole scope of what they’re dealing with. So that gives them an idea of what’s ahead of them and how they’re going to have to face certain things or changes in their loved one” (Support Staff site 3).

#### From hierarchies to building a team approach

All the staff were seen to have a role to play in ACP, yet in practice, charge nurses and doctors take the lead, and the other staff are seen as useful in informing or triggering them to activate the conversation. Participants highlighted the hierarchical nature of LTCH workforce limiting the interdisciplinary approach to ACP. Many PSWs and other support staff experienced moments where residents shared their future care values and wishes. Yet, many do not follow up these discussions with other members of the LTCH team as they do not feel it is their place, due to lack of team approach. This PSW articulated their experience below:

So if a resident or a family member were to come to me about that kind of conversation…I would direct her to the administrator. We would refer them to the Charge Nurse, and they would follow the chain of command from there. (PSW site 3)

At the same time, all categories of staff also shared a collective appreciation and enthusiasm to function as part of a team to move forward the ACP conversations. Though there are existing hierarchies, many asserted that informal ACP conversations are common, especially among residents and PSWs and/or support staff. Therefore, these staff should be empowered and trained to not only effectively engage in such conversations but be supported to contribute as a valued team member. One physician shared their aspirations.

I think it has to be like a team, the whole staff has to be a team. So that everybody’s input…because the PSWs are the primary caregivers so their input with every shift needs to be heard or if they see changes in the resident. They are the people that are seeing the resident the most.” (Physician site 2)

As such participants emphasized the importance of documenting all communication, not just formal conversations so they are accessible for the whole care team. Most staff acknowledged that ACP is not only about the EOL care planning, but also about what residents want to accomplish during their stay in the LTCH. ACP should involve the type of activities and people that give residents joy and those they want to refrain from. Participants also explained that ACP communication should expand beyond medical and physical needs, and should include emotional, spiritual and comfort needs. The importance of an interdisciplinary approach was discussed, and all participants agreed that ACP communication requires a team approach to learn and honor residents’ values and wishes. As well, in coordinating their plan of care that should involve residents and their family care partners. This support staff described their take on the interdisciplinary nature of ACP and overall person centered care for LTCH residents:

Advanced Care Planning is not just about medication or programs; it is also about type of food that you want…Or how you might want your room to be set up or whatever. Or if you want your family to be there. So it’s an interdisciplinary approach. So, whatever is put in the care plan we all have to be aware of it, we all have to be a part of the implementation along with family and the resident. (Support Staff site 2)

Fundamentally, all participants recognized the critical importance of ACP discussions and realized complexities surrounding them. Staff described the importance of implementing an interdisciplinary team approach to incorporate ACP as part of caring for residents. Though each category of staff saw their role in carrying out ACP conversations, hierarchical nature of LTCH staffing limits collaborative practice and impacts staff’s level of confidence and comfort for broaching the subject with residents, family, and other staff.

## Discussion

This study sought to explore the perspectives of LTCH staff on engaging in ACP discussions with residents they care for. A palliative approach that incorporates ACP should be integrated with ongoing chronic disease management [55], yet it is not a norm practiced in most LTCHs [56]. Evidence indicates that the use of invasive life-sustaining treatments has doubled for LTCH residents including those with advanced dementia. These invasive treatments, such as mechanical ventilation, have demonstrated insignificant influence on resident mortality [57] but poor impact on their wellbeing and dignity [20]. Whereas, ACP conversations in LTCHs have led to positive outcomes such as reduction in hospital transfers, care partner satisfaction, wish concordant EOL care and better pain and symptom management [58]. We describe LTCH staff’s perceptions in relation to their understanding of the ACP process, experiences related to formal and informal engagement in ACP conversations, barriers and facilitators to ACP, and aspirations for implementing an interdisciplinary team approach to bring forth residents’ wishes for comprehensive and informed future care. The findings of this study provide important contributions to our understanding of the complexities surrounding ACP implementation as a standard of practice in the LTCHs.

ACP is an important component of integrating a palliative approach in LTCHs. However, ACP requires the resident have decision making capacity to engage in thinking about their values and wishes for future care and sharing these with those they trust [59]. One of the key findings of this study was staff’s uneasiness about exclusion of capable residents in ACP discussions while devising care plans with family care partners and/or proxy decision makers. Many staff shared their frustrations with discordance between what residents desired and the decisions made by families. In many cases, residents repressed their wishes to not hurt their families. At the same time, family care partners may be protecting their residents from psychological distress frequently associated with ACP, by not involving them in the ACP discussions. Engagement of capable residents in the ACP discussions can help them exercise their autonomy regarding their own future care. Future studies should explore perspectives of care partners and residents related to their autonomy to engage in ACP, congruence in wishes between residents and care partners and psychological distress with ACP conversations. It is crucial that residents and families are involved in care planning as a unit, where residents feel safe and supported to share their wishes with family and staff. Complex interactions within and between families were identified as key challenges to ACP engagement. It is important therefore that ACP standards are created that promote residents and families to learn about each other’s viewpoints, while keeping residents’ wishes at the core. Previous studies have demonstrated engaging in ACP discussions together served to be therapeutic for both [60, 61]. The family care partners might have genuine issues needing to be addressed, and the role of LTCH staff would be to consider needs of both and facilitate discussions while promoting optimal involvement of residents [62]. This also speaks to the significance for training on communication rather than simply knowledge about illness trajectories and care options at the EOL.

One of the critical findings of this study relates to a lack of ACP conversations prior to admission in the LTCHs, by which time many residents have already lost cognitive abilities to be able to engage in these discussions. Lack of knowledge about the residents’ values and wishes can result in life-sustaining measures at the EOL they may not desire or benefit from. The situation becomes more complex when residents have no friends and families to share in the ACP process. It is a missed opportunity when primary care providers and other clinicians in community and acute care sectors fail to initiate ACP conversation at the outset of the chronic disease trajectory. Given that ACP is a dynamic process, there is opportunity to discuss goals of care with any change in illness trajectory such as worsening of dementia or transition to LTCH [63]. As such, admission to LTCH can serve as a trigger to engage residents and their care partners in ACP. However, if residents have lost their decision-making capacity, clinicians should engage in a goals of care conversation with residents’ proxy decision makers including the PG. Though goals of care conversations are not a common practice [64], they demand honest disclosure of residents’ prognosis to guide the EOL care planning conversation and help in the decision-making process. Prior ACP discussions have shown to increase care partners’ decision making confidence [61, 65]. Future studies can examine if open and thorough goals of care discussions also impact decision-making confidence in proxy decision makers.

While all staff were seen to have a role to play in ACP there was a hierarchical nature to what each role should be doing. This stands in contradiction to literature on interdisciplinary collaboration required for a successful implementation of ACP initiatives [66]. Our findings highlighted that, nurses and physician were viewed as having authority to initiate and facilitate formal ACP communication, whereas PSWs and support staff were suited to identifying residents and families who could benefit from an ACP conversation. Participants also perceived that PSWs should be better utilized in ACP communication, as they often know residents and families better than other staff members. However, this study and others have noted that these staff report lacking sufficient knowledge, skills, and comfort to participate in ACP [17, 23–27]. PSWs and support staff are unregulated and typically help residents with personal care and activities of daily living according to an established care plan. Given that these direct care staff have the highest proportion than other staff members in the LTCHs, there is a need to include them in ACP as important team members. These staff can be systematically trained and empowered through education and encouragement to support ACP by engaging with residents and family care partners in informing about ACP initiatives, facilitating ACP conversations and documenting these encounters for the continuity of care. Communication tools such as SBAR (Situation, Background, Assessment, Recommendation) [67], SPIKES (Setting, Perception, Invitation, Knowledge, Emotions, Strategy and Summary) [68] and SICG (Serious Illness Conversation Guide) [69] should be tested for optimizing staff’s confidence and practice. Furthermore, each of the team members’ observations and communication need to be valued and incorporated in the residents’ care plan. In essence, there is need for revitalizing care in LTCHs through interdisciplinary care practices, clarification of role descriptions, optimized staffing, capacity building of each category of staff including external consultants and commitment from the LTCH leadership for such care.

## Limitations

It is important to note limited transferability of findings of this study to other settings and contexts. This study was designed to describe and understand the local context; therefore, the findings need to be applied with caution to contexts other than urban Toronto and Hamilton, Ontario LTCHs. Also, the purpose of the current study was to explore perceptions about ACP based on perspectives of interdisciplinary staff in LTC homes. In the future, we can attempt to examine and compare perspectives from different staff members with a larger sample size for each of the staff category.

## Conclusion

The findings from this study build on a growing body of research illustrating the need to improve LTCH staff engagement in ACP communication. All participants within our study expressed support for ACP communication in the LTCH and the need for open lines of formal and informal interdisciplinary communication, including the resident and family care partners. Providing staff with ACP communication training, supporting interprofessional collaboration, and the developing and implementing resources and processes for early ongoing ACP communication need to be prioritized in LTCHs to support the engagement of a wide range of staff.

## Supplementary Information

**Additional file 1.** Functions of different categories of staff

**Additional file 2.** Focus Group Guide

**Additional file 3.** Themes, subthemes and additional illustrative quotes

## Data Availability

The data generated and analysed during the current study are not publicly available due a potential breach of privacy of participants and settings they were recruited from. But it can be made available from the corresponding author on reasonable request.

## Abbreviations

LTCH: Long-term care home
ACP: Advance care planning
EOL: End-of-life
HCP: Health care provider
PG: Public guardian
POA: Power of attorney
PSW: Personal support worker
RPN: Registered practical nurse
LEAP: Learning Essential Approaches to Palliative Care
COPD: Chronic obstructive pulmonary disease; SS Support staff
SBAR: Situation Background Assessment Recommendation
SPIKES: Setting perception invitation knowledge emotion summary
SICG: Serious illness conversation guide
