# Supplementary material for: Interdisciplinary staff perceptions of advance care planning in long-term care homes: a qualitative study
Source: BMC Palliat Care. 2022 Jul 15;21:127. doi: 10.1186/s12904-022-01014-2 (PMC9284816; doi:10.1186/s12904-022-01014-2)
Supplement: Supplementary file 1 — Additional file 1. Functions of different categories of staff [file 12904_2022_1014_MOESM1_ESM.docx]

Additional File 1. Functions of Different Categories of Staff

| Category of Staff | Functions | Role in ACP | Qualifications |
| --- | --- | --- | --- |
| Nursing staff (RN & RPNs)  Regulated/registered | - Train and delegate non-registered staff on different skills such as administration of topical medications and supervise them. - Medication management - Perform physical and mental assessments - Collaborating with healthcare professionals including physicians - Take on a leadership role - Delivery of skilled procedures according to the College of Nurses of Ontario Standards of Practice | - RNs communicate with family of residents and provide updates on their health and well-being, as well as discuss the ongoing care and what the family can expect in the future - Organize and conduct care conferences for ACP and goals of care conversations | - Current certificate of registration with the College of Nurses of Ontario |
| PSW  Unregulated | - Provides personal support services to assist with the activities of daily living, including personal hygiene services, and includes supervision in carrying out those activities - Perform tasks delegated by a registered health professional - Assist with medication and medication reminders - Perform light housekeeping duties (Ontario Personal Support Workers Association) | - Informal conversation with residents during personal care and assisting with other activities of daily living. | - Successful completion of a PSW program. - The program must be a minimum of 600 hours in duration, counting class time and practical experience time |
| SS  Unregulated | - Assist with basic activities of daily living with other staff and recreational interventions. | - Informal conversation with residents during personal care and assisting with other activities of daily living. |  |
| PG  Appointed by the court | - A guardian of the person is someone appointed by the court to make personal care decisions on behalf of a mentally incapable adult - A guardian of the person can make personal care decisions for health care, nutrition, shelter, clothing, hygiene and safety | - Do not initiate ACP conversation but assist staff with decision making when called for. | The Lieutenant Governor in Council may appoint a member of the bar of Ontario to be Public Guardian and Trustee |

RN Registered nurses; RPN Registered practical nurses; PSW Personal support worker; SS Support staff; PG Public guardian
