# Supplementary material for: Interdisciplinary staff perceptions of advance care planning in long-term care homes: a qualitative study
Source: BMC Palliat Care. 2022 Jul 15;21:127. doi: 10.1186/s12904-022-01014-2 (PMC9284816; doi:10.1186/s12904-022-01014-2)
Supplement: Supplementary file 2 — Additional file 2. Focus Group Guide [file 12904_2022_1014_MOESM2_ESM.docx]

Additional File 2. Focus Group Guide

| 1. What do you think about the idea of advance care planning (ACP)? 2. What is most important when discussion end-of-life care with residents and their families? 3. What is most important to discuss during ACP conversation? 4. How do see ACP implemented in your long-term care home? 5. What is your role in implementing ACP? 6. How often ACP conversations are revisited? 7. What do you think is done particularly well in implementing ACP conversations? 8. How do you communicate with residents and families about ACP? 9. What do residents want to know (based on your experience)? 10. What are the challenges of initiating ACP? 11. What are the roles of different team members in implementing ACP conversations? 12. How do you communicate ACP discussions amongst the team members? |
| --- |

ACP Advance care planning
