# Supplementary material for: Interdisciplinary staff perceptions of advance care planning in long-term care homes: a qualitative study
Source: BMC Palliat Care. 2022 Jul 15;21:127. doi: 10.1186/s12904-022-01014-2 (PMC9284816; doi:10.1186/s12904-022-01014-2)
Supplement: Supplementary file 3 — Additional file 3. Themes, subthemes and additional illustrative quotes [file 12904_2022_1014_MOESM3_ESM.docx]

Additional File 3. Themes, Subthemes and Additional Illustrative Quotes

| **Themes** | **Subthemes** |  |
| --- | --- | --- |
| Ongoing nature of ACP | - Right time to initiate ACP discussions is when the person can participate | - So it really has to be done long before Long Term Care of today anyway. Because the residents are coming in much sicker, much more deteriorated than they used to. (Nurse 1, site 1) - It is about what was done prior to admission here. If we have a one hundred year old person who never had any discussions or the family never had discussions about what’s going to happen, I mean it’s inevitable for it to happen. But then it’s quite difficult for them to react to our discussions here. So the discussion should take place prior to being admitted to Long Term Care…two heart surgeries and the discussion never took place. So now they’re here. (Nurse 7, site 1) - We are all different and we all want different things for ourselves. And that is why you know while we are capable of expressing those wishes we should be able to do that and those wishes become part of a chart. But I don’t think it’s a good idea to wait until the last moment…I think that if the family physicians see their patients deteriorating, they should be the ones to start the conversations. Because they know their patients better. They know their families better. (Physician site 4) |
|  | - ACP discussions can make transition to EOL more seamless | - And that’s the important thing when we talk to families is to remind them. You may not have had the discussion in the last year but throughout that person’s life have they ever said anything to you about, I don’t want to be kept alive by machines. Like those kinds of conversations. You have to sometimes help them recall those events in their lives. (Nurse 1, site 1) - if you’re going to diagnose somebody with Dementia, you know that they’re going to progress. So the conversation has to happen a little bit earlier in the process of disease and not later. But all the time we have to kind of try to find out from the family what the wishes of your patient would have been. You know try to get to know your patient right? What they would want you to do for them. So it’s a little bit challenging (4 physician) - I think one would be the person concerned like the one who is experiencing it. Her choice or his or her choice of how they would like their end of life. I think that would be the best thing to talk about. Not the family’s choice or some of the staff choice.(RPN) - Advanced Directives are supposed to be a person’s own wishes…not from their substitute decision maker or family. And if we don’t know what their wishes are then we can’t really sort of speak to that on their behalf. But if we know what their wishes are or if have any friends or family that can help us kind of learn what the person’s wishes might be…then we try to gather that or we try to encourage facilities to do that. (PG) |
|  | - ACP should be holistic to guide EOL care | - So we just started implementing My Wishes (with recreational staff to talk about preferences), residents that are more able to you know dictate what they need at the end or what would they like to see happen and stuff so…(Nurse , site 1) |
| Complexities around ACP conversations | - Identifying residents’ values and wishes when no former ACP | - And that’s the important thing when we talk to families is to remind them. You may not have had the discussion in the last year but throughout that person’s life have they ever said anything to you about, I don’t want to be kept alive by machines. Like those kinds of conversations. You have to sometimes help them recall those events in their lives.(Nurse, site 1) |
|  | - Navigating divergent resident and family perspectives | - Sometimes there’s tremendous conflict and you know I’ve had situations where a resident or patient has a quality of life and wants to die, is ready to die and family can’t let go, won’t let go. I’ve had situations where one family member is hanging on no matter what, and the other family member is much more realistic and understanding. (Physician site 1) - if resident comes forth to express their wishes such as for pain control at end of life, then staff would inform the family of their wishes, our role in that discussion would be as the advocate for the resident." (Nurse 1, site 1) - “Someone could think oh I think this is their best wishes but another child could think something else. So it’s definitely important to talk about and even for the family to sit down and talk about it separately, maybe they want to do that privately and then come back and have another meeting to discuss it again.” (SS4) - Ultimately it’s up to the family, power of care to decide...But I think as our role, my role and the staff role is to provide them with any information they need to make the better decision. That’s all. |
|  | - Staff’s lack comfort to broach ACP conversations | - Because I would actually want somebody who actually has some kind of concrete proof. Because this is my visual observation but I want, I think the nurse or the doctor should be able to say, well their heart rate is not the same, their blood pressure is not the same, their having heart beat problems. Even if I tell them your Mother is declining I can not elaborate for them. …So I would much rather the nurse or the Doctor say that to the family, not me - I would say having all of our physicians on the same page with respect to Palliative Care, because everyone is unique. Some residents are admitted relatively strong and then their condition changes and I’ve been to Team Care Conferences where physicians take different approaches to speaking about Palliative Care. Some were very open about it and direct. Others don’t really address it. So I know we took that Leap training for Palliative Care and we were talking about getting the doctors to take that as well. I think them all having the same baseline understanding and be on the same page so that they can address the issue. Because I think they, you know…admission interviews is a great time to mention that. (Nurse, Site 1) |
| Aspirations for ACP becoming a standard of care in LTCHs across all disciplines | - Prioritizing ACP as a standard of care | - We have the pamphlet and information at the front desk and then upon admission as well. I think we need to focus on getting those families to have those discussions as hard as they may be. Because I find that’s what the barriers are a lot of the time, that the families are reluctant to speak about it. (SS3) |
|  | - Need for training and capacity-building for staff to support residents and families in ACP | - if we have something like a reference. Even if it’s questionnaires. Because then you can guide yourself as well. Because it’s a lot to remember and it’s stressful. So if you can say if this happens, number two, number three and it’s so much easier and everybody will be on the same page. (Nurse 6, site 1) - So what it means to me is having a road map going forward as to how to deal with decline in patients. (1 physician) - 4 PSW: P: Yes I guess it’s really important, especially to PSW that just got hired, because it’s very important to know the preferences and the needs of the residents. Especially when they reach the Palliative stage. It’s easy to take care, the personal care you do every day but when the time comes that they’re dying, we need to know how we approach the family. It’s very important. I guess to have session like this, you learn from each other and also from…what I’m going to say…from the management to give PSW a session like this more often. - Sometimes it might be helpful to get PSWs involved a little bit just because I’ve seen scenarios where you know I’ve had a discussion with families about a resident who is declining, not eating, and then there’s a PSW saying you know we’re not doing enough, we need to give her supplements and we need to do this and we need to do that....General education about what it is, not necessarily how to facilitate but it doesn’t hurt to know what’s going on. Not on a higher level. More of a summary versus specific details and facilitating. (Nurse, Site 1) |
|  | - From hierarchies to building a team approach | - “I was doing a visit and he just was talking about how he was afraid of what was happening to him. And we just sat and talked, because he was from a particular faith so we just opened the Bible and we read a bit and we talked about what we read and carried the conversation from there” (SS3) - Would these informal conversations still be considered…would it still be implemented as part of the advanced care plan? So let’s say for example a PSW had a conversation with a resident and they were talking about some aspect of their advanced care plan that they wanted to change...Absolutely that’s part of the advanced care planning right. (4 Physician) - “I think it has to be like a team, the whole staff has to be a team. So that everybody’s input…because the PSWs are the primary caregivers so their input with every shift needs to be heard or if they see changes in the resident. They are the people that are seeing that resident the most” (6 Physician) |
